# Heisenberg-limited single-mode quantum metrology in a superconducting circuit

**DOI:** 10.1038/s41467-019-12290-7

**Published:** 2019-09-26

**Authors:** W. Wang, Y. Wu, Y. Ma, W. Cai, L. Hu, X. Mu, Y. Xu, Zi-Jie Chen, H. Wang, Y. P. Song, H. Yuan, C.-L. Zou, L.-M. Duan, L. Sun

**Affiliations:** 1 Center for Quantum Information, Institute for Interdisciplinary Information Sciences, Tsinghua University, 100084 Beijing, China; 2 Department of Physics, University of Michigan, Ann Arbor, MI 48109 USA; 3 Key Laboratory of Quantum Information, CAS, University of Science and Technology of China, 230026 Hefei, Anhui China; 4 Department of Mechanical and Automation Engineering, Chinese University of Hong Kong, Shatin, New Territories, Hong Kong, China

**Keywords:** Quantum metrology, Single photons and quantum effects

## Abstract

Two-mode interferometers lay the foundations for quantum metrology. Instead of exploring quantum entanglement in the two-mode interferometers, a single bosonic mode also promises a measurement precision beyond the shot-noise limit (SNL) by taking advantage of the infinite-dimensional Hilbert space of Fock states. Here, we demonstrate a single-mode phase estimation that approaches the Heisenberg limit (HL) unconditionally. Due to the strong dispersive nonlinearity and long coherence time of a microwave cavity, quantum states of the form $\left( {\left| 0 \right\rangle + \left| N \right\rangle } \right)/\sqrt 2$ can be generated, manipulated and detected with high fidelities, leading to an experimental phase estimation precision scaling as ∼*N*^−0.94^. A 9.1 dB enhancement of the precision over the SNL at *N* = 12 is achieved, which is only 1.7 dB away from the HL. Our experimental architecture is hardware efficient and can be combined with quantum error correction techniques to fight against decoherence, and thus promises quantum-enhanced sensing in practical applications.

## Abstract (web summary)

Reaching a quantum advantage in metrology usually requires hard-to-prepare two-mode entangled states such as NOON states. Here, instead, the authors demonstrate single-mode phase estimation using Fock states superpositions in a superconducting qubit-oscillator system.

## Introduction

High precision measurement is one of the main driving forces for science and technology, and the interferometer based on the coherent interference effect is one of the most extensively used tools^1–6^. Two-mode interferometers, in particular, are widely used to precisely measure the phase difference between the two modes induced by certain physical quantities. For example, the two-mode atomic Ramsey interferometer that manipulates the superpositions of two internal states of an atomic ensemble^7^ has been used in various applications, such as clock^8,9^, gravimeter^10^, and gyro^11^. Similarly, by separating photons into two spatial modes, two-mode photonic Michelson interferometers have been extensively used in gravitational-wave observatory^5^, optical coherence tomography^12^, and spectrometry^13^. Recently, quantum metrology^14–16^, which makes use of quantum mechanical effects, such as entanglement, has gained a lot of attention in the two-mode interferometers, as it can achieve measurement precisions beyond the classical limit. In the applications of quantum metrology, highly entangled states, such as the Greenberger–Horne–Zeilinger state of an atomic ensemble^17,18^ or the NOON state of optical interferometer^19,20^, are essential. However, to prepare these exotic quantum states, non-local operations are required. In addition, the optimal measurements are also typically highly non-local. These pose significant challenges for practical applications of quantum metrology.

Instead of exploring quantum entanglement in the two-mode interferometer, quantum sensors with a single mode are of great interest. For example, based on a single bosonic mode, quantum metrology schemes have been proposed^21,22^ by taking advantage of the infinite-dimensional Hilbert space of Fock states. Such single-mode quantum sensors hold the advantages of hardware efficiency, compactness, and robustness against non-local perturbations. Similarly, by exploring the large angular momentum states, a high precision electrometer beating the shot-noise limit (SNL) is promising with a single atom, as demonstrated in refs. ^23,24^. Alternatively, by utilizing coherence and implementing phase estimation algorithms, a quantum-enhanced magnetometry was recently demonstrated with a single artificial atom^25–27^, with a precision approaching the Heisenberg limit (HL).

In this paper, we implement the single-mode photonic quantum metrology with a superconducting qubit-oscillator system^28^ and demonstrate an unconditional phase estimation approaching the HL. For a single mode, the phase can be measured based on the photon number-dependent phase accumulation. By preparing the superpositions of Fock states as $\left| {\Psi \left( N \right)} \right\rangle = \left( {\left| 0 \right\rangle + \left| N \right\rangle } \right)/\sqrt 2$ up to *N* = 12, we demonstrate a phase estimation precision that scales as $\delta \tilde \theta \sim N^{ - 0.94}$ and approaches the HL. At *N* = 12, $\delta \tilde \theta$ corresponds to an enhancement of $20{\mathrm{log}}_{10}(\delta \tilde \theta _{{\mathrm{SNL}}}/\delta \tilde \theta )\,{\mathrm{dB}} = 9.1\,{\mathrm{dB}}$ over the SNL $\delta \tilde \theta _{{\mathrm{SNL}}}$. Envisioning future applications in the optical regime with microwave-to-optical transduction, we also realize a measurement scheme that is easy to implement in optics and only uses displacement operations and photon counting. Under this restricted measurement scheme, a sub-SNL precision, which scales as $\delta \tilde \theta \sim N^{ - 0.62}$, is also achieved.

## Results

### Theory of optimal sensing and experimental architecture

**Fig. 1:**
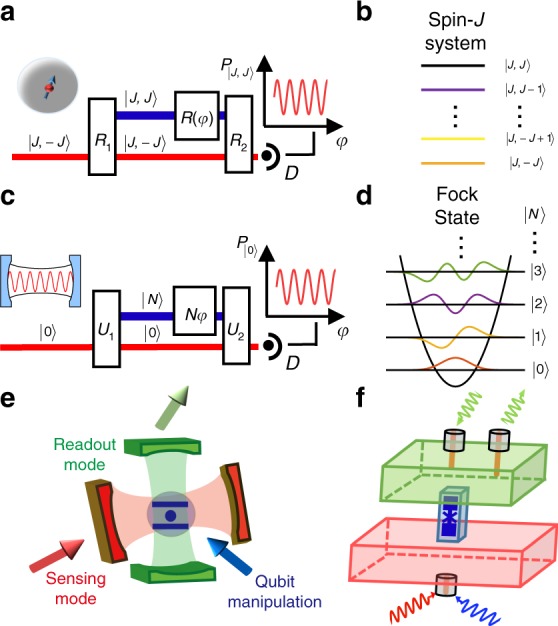
Single-mode quantum metrology architectures. **a**, **b** Single-atom Ramsey interferometer with a total angular momentum number *J*. The best precision can be achieved by using the superposition of |*J*, −*J*〉 and |*J*, *J*〉 with the maximum variance of angular momentum. **c**, **d** Single-mode Ramsey interferometer for photons/bosons with an optimal precision achieved by the superposition of Fock states with the maximal variance of photon numbers for a given mean photon number. **e**, **f** Schematic illustrations of the single-mode sensing architecture and the experimental circuit quantum electrodynamics system. A single qubit couples to two photonic cavity modes, with the two modes serving as the sensing mode and the ancillary qubit readout mode, respectively. The two boxes represent the microwave cavities, between which the ancillary superconducting qubit on a chip is located in a waveguide trench

According to the quantum Cramér–Rao bound^29^, the estimation precision of parameter *θ* encoded in the state |*ψ*(*θ*)〉 = e^−*iθH*^|*ψ*〉 is lower bounded as $\delta \tilde \theta \ge \frac{1}{{2\Delta H}}$, where $\delta \tilde \theta$ is the standard deviation of an unbiased estimator $\tilde \theta$, and (Δ*H*)^2^ = 〈*ψ*|*H*^2^|*ψ*〉 − 〈*ψ*|*H*|*ψ*〉^2^ is the variance of the Hamiltonian *H* with the initial probe |*ψ*〉. The quantum states with a maximum variance therefore are optimal for the single-mode sensing, i.e. the equal superpositions of the eigenstates of *H* corresponding to the extreme eigenvalues are the most preferable quantum states. For example, as illustrated in Fig. 1a, b, an atom prepared in the equal superposition of angular momentum states |*J*, −*J*〉 and |*J*, *J*〉 has maximal sensitivity to external field (*H* = *J*_*z*_ and *J*_*z*_ is the angular momentum operator). Recently, a high precision electrometer, using the Schröinger cat state of large angular momentum states to enhance Δ*H*, has also been demonstrated to beat SNL^23,24,30^. Similarly, the phase precision with a single bosonic mode would be enhanced by using the state $\left| {\Psi \left( N \right)} \right\rangle = \left( {\left| 0 \right\rangle + \left| N \right\rangle } \right)/\sqrt 2$ (Fig. 1c, d), since it has the maximum variance for $H = a^\dagger a$ (*a* is the bosonic operator of the sensing mode), given a mean photon number (average energy). Such a maximum variance state (MVS) can in principle achieve the HL precision $\delta \tilde \theta = 1/N$ with $\sqrt N$ times enhancement over the SNL.

As schematically illustrated in Fig. 1e, f, our experiment is carried out with a superconducting system consisting of a transmon qubit dispersively coupled to two three-dimensional cavities^31–33^. The long-lived cavity serves as the sensing mode; the transmon qubit as an ancilla assists the preparation, manipulation, and detection of the photonic states in the sensing mode; the short-lived cavity is employed for a high-fidelity readout of the qubit state. The Hamiltonian of the qubit-oscillator system is $H = - \hbar \chi _{{\mathrm{qs}}}|e\rangle \langle e|a^\dagger a$^28^, where |*e*〉 is the excited state of the qubit (the ground state is |*g*〉), and *χ*_qs_ reflects the dispersive interaction strength between the qubit and the mode (Supplementary Note 1). In our system, *χ*_qs_/2*π* = 1.90 MHz is much stronger than the decoherence rates of the qubit and the sensing mode, thus allows full control of the photonic quantum state^32–36^.

### Preparation of the MVS states

**Fig. 2:**
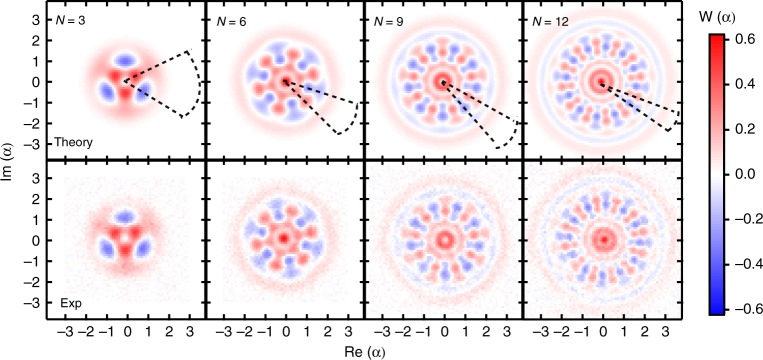
Wigner functions of the maximum variance states. Theoretical (top panel) and experimental (bottom panel) results for $\left( {\left| 0 \right\rangle + i\left| N \right\rangle } \right)/\sqrt 2$ with state fidelities of 0.94, 0.92, 0.83, 0.70 for *N* = 3, 6, 9, 12, respectively. The measured state preparation fidelity decays with *N*, mainly attributed to the larger probability of photon loss and the worse reconstruction measurement to obtain the Wigner functions for larger *N*. The measured range of the real and imaginary parts of *α* is [−3.0, 2.9] for *N* = 3 and 6, [−3.6, 3.5] for *N* = 9, and [−3.9, 3.8] for *N* = 12. The angles of the dashed sectors indicate that the sensitivity of phase estimation scales as 1/*N*

In our experiment, the probe states of the sensing mode are deterministically created by implementing a qubit-assisted unitary operation on the mode based on carefully calibrated experimental parameters. The unitary operation is realized through the so-called gradient ascent pulse engineering method^37,38^, which is an optimization algorithm designed to numerically find pulses that most accurately realize a unitary operation and has been widely used for creating cat states^32,35^ and other superpositions of Fock states^33^. With numerically optimized control pulses, the probe states |Ψ(*N*)〉 with *N* = 1, 2, …, 12 are prepared faithfully. Compared with the previous scheme that prepares superpositions of Fock states by climbing the Jaynes–Cummings ladder step-by-step^39^, our one-step approach allows arbitrary state preparation with higher fidelity and shorter operation time. In Fig. 2 the experimentally measured Wigner functions (bottom panels) of the typical MVSs are plotted, agreeing well with the ideal ones (top panels). In the phase-space, there are interesting periodic fringes in the polar direction with *N*-fold rotational symmetry for |Ψ(*N*)〉. As the rotation of the Wigner function by *θ* corresponds to the phase operation $U\left( \theta \right) = {e}^{i\theta a^\dagger a}$ on the oscillator, the enhanced measurement precision by the MVS can be intuitively explained: because of the fine fringe features, |Ψ(*N*)〉 would be rotated to an orthogonal state when the phase *θ* = *π*/*N*, the measurement precision with the MVS is thus proportional to *N*, instead of $\sqrt N$.

### Optimal single-mode sensing scheme

**Fig. 3:**
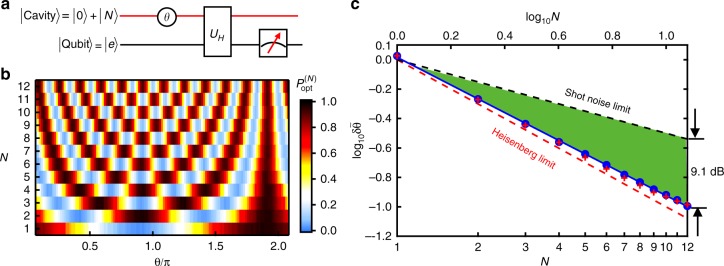
Optimal single-mode sensing scheme. **a** Quantum circuit. **b** The measured probability $P_{{\mathrm{opt}}}^{(N)}$ of projecting to |Ψ(*N*)〉 as a function of the phase *θ* in the optimal scheme for different *N*. The outcomes are obtained with 10^6^ repetitions of the experiment. **c** Quantum advantage of the optimal scheme. Blue dots are experimental results. The blue solid line is a linear fit and gives $\log _{10}\delta \tilde \theta = - 0.94\log _{10}N + 0.016$ with the precision scaling *N*^−0.94^ approaching the Heisenberg scaling (*N*^−1^). The small offset 0.016 in the log–log scale of the precision is dominantly due to the imperfections of the qubit readout process, where the spontaneous decay of the qubit gives wrong indication of the cavity state and lowers the contrast of the Ramsey interference. The red crosses are the results from numerical simulations including the decoherences of our system, in good agreement with the measured data. The error bars, obtained through the standard deviations of *A* and *B* in fitting the data in **b**, are smaller than marker sizes. The black and red dashed lines are the theoretically calculated SNL and HL, respectively. Green region represents the experimental results that surpass the standard limit by about 9.1 dB at *N* = 12

Figure 3a depicts the experimental circuit for the optimal sensing scheme with a Ramsey-like interference (Fig. 1c), which can attain the ultimate HL for the single-mode sensing. After an initialization process, the cavity is prepared in |Ψ(*N*)〉 while the qubit ends up with |*e*〉. The phase operation *U*(*θ*) on the sensing mode can then be generated after the system evolves for a time period *τ*, where *θ* = −*χ*_qs_*τ*. Then, a unitary *U*_*H*_ is implemented to rotate $\left| {{\mathrm{\Phi }}_ + } \right\rangle = \left| e \right\rangle \left( {\left| 0 \right\rangle + \left| N \right\rangle } \right)/\sqrt 2$ to |*g*〉 |0〉 and $\left| {{\mathrm{\Phi }}_ - } \right\rangle = \left| e \right\rangle \left( {\left| 0 \right\rangle - \left| N \right\rangle } \right)/\sqrt 2$ to |*e*〉 |0〉. Finally, the ancillary qubit is projectively measured on |*g*〉, giving projection of *U*(*θ*)|Ψ(*N*)〉 onto |Ψ(*N*)〉 with the ideal probability oscillation (Supplementary Note 2)

1 $$P_{{\mathrm{opt}}}^{(N)} = \left( {1 + {\mathrm{cos}}N\theta } \right)/2$$

The experimental results of the optimal scheme $P_{{\mathrm{opt}}}^{\left( N \right)}$ are shown in Fig. 3b. As intuitively expected from Fig. 2, the period of the Ramsey interference fringes reduces with *N* and the contrast of the fringes are nearly ideal. By fitting the experimentally measured probability with *P*^(*N*)^(*θ*) = *A* + *B* cos(*Nθ*), where *A*−*B* and *B* represent the detected background and the contrast of the Ramsey interference fringes, respectively, the phase estimation precision can be inferred as

2 $$\delta \tilde \theta = \frac{{\sqrt {P^{(N)}\left( \theta \right)\left( {1 - P^{(N)}\left( \theta \right)} \right)} }}{{\left| {\frac{{\partial P^{(N)}\left( \theta \right)}}{{\partial \theta }}} \right|}} = \frac{{\sqrt {A\left( {1 - A} \right)} }}{{NB}}.$$

Figure 3c shows the results of $\delta \tilde \theta$ (blue dots) as a function of *N* in a logarithmic–logarithmic scale. Clearly, the optimal scheme beats the SNL, with the green region representing the experimental results that surpass the SNL with a maximum precision enhancement of 9.1 dB at *N* = 12, which is only 1.7 dB away from the ultimate HL. The results demonstrate the quantum advantage of our single-mode sensing unambiguously. The obtained precision scales as *N*^−0.94^, approaching the Heisenberg scaling (*N*^−1^). The slight deviation mainly attributes to the *N*-dependent imperfections including the larger operation errors for larger Hilbert space of Fock states (errors in the control pulse and parameter uncertainties) and higher probability of photon loss.

The demonstrated optimal scheme can be utilized in practical sensing applications. For example, if there is another microwave signal coupling with the qubit with unknown amplitude or frequency but largely off-resonant with the qubit-oscillator system, the frequency of the sensing mode would be shifted due to the cross-Kerr effect mediated by the qubit. This effect is equivalent to an AC Stark-effect-induced shift of the sensing mode. This frequency shift can cause an accumulated phase on |*N*〉 with respect to |0〉, which can be detected by the presented optimal sensing scheme. In turn, we can estimate the frequency or amplitude of the unknown microwave signal.

### Hybrid single-mode sensing scheme

**Fig. 4:**
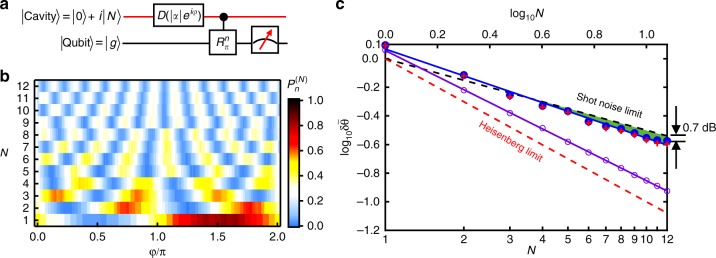
Hybrid single-mode sensing scheme. **a** Quantum circuit. The initial state |0〉 + *i*|*N*〉 corresponds to an arbitrarily chosen phase *θ* = *π*/2*N* to demonstrate the idea of the phase estimation. The detection is realized by a combination of a displacement of the photonic mode [*D*(*α* = |*α*|e^*iφ*^)] and selective photon number (|*n*〉 state) detection enabled by the ancillary qubit through its dispersive interaction to photons. **b** The measured probability $P_n^{(N)}$ of projecting to the photon number state |*n*_opt_〉 as a function of *φ* in the displacement operation, with the optimal *n*_opt_ for each *N* being numerically obtained. **c** Quantum advantage for the hybrid scheme. Blue dots are experimental results. The blue solid line is a linear fit and gives $\log _{10}\delta \tilde \theta = - 0.62\log _{10}N + 0.068$. The red crosses are the results from numerical simulations including the decoherences of our system, in good agreement with the measured data. Green region represents the experimental results that surpass the standard limit by about 0.7 dB at *N* = 12. Purple circles show the achievable precision for the hybrid sensing scheme with a fit $\log _{10}\delta \tilde \theta = - 0.91\log _{10}N + 0.057$, provided all photon numbers can be detected. The error bars, obtained through the standard deviations of $A_n^{(N)}$ and $B_n^{(N)}$ in fitting the data in **b**, are smaller than marker sizes. The scaling *N*^−0.62^ (*N*^−0.69^ for an ideal experiment) can still beat the standard scaling *N*^−0.5^ due to the initial MVS. The offset of the hybrid scheme is mainly due to the imperfect photon number detection

By utilizing the recently developed high-efficient bidirectional microwave-to-optical quantum transduction^40,41^, our scheme with the MVS can also be employed for the optical metrology. However, the Ramsey-like measurement is very challenging in optical domain due to the limited capability of deterministic quantum state manipulation of optical photons. We thus propose a hybrid sensing scheme, as shown in Fig. 4a, by employing a measurement scheme that only uses easy operations in the optical domain, such as displacement operation and photon counting^42^.

Envisioning the application of such a hybrid scheme, we simulate the scheme in our superconducting system with the restricted measurement. It is worth noting that different from photon counting in the real optical system^42^, the measurement through the ancillary qubit in the superconducting system can only obtain a binary output, i.e. a result of whether the photon number is *n* or not. So, the outcome of the measurement has the probability $P_n^{(N)} = \left| {\langle n|D\left( \alpha \right)U\left( \theta \right)\left| {\Psi \left( N \right)} \right\rangle } \right|^2$, where *D*(*α*) is the displacement operator. We optimize the parameters *α* and *n* for each MVS to maximize the measurement precision (Supplementary Note 2).

The experimental results for the simulated hybrid scheme are summarized in Fig. 4b. Although the fringe period reduces with *N* similar to that in the optimal scheme, the contrast for the hybrid scheme reduces with *N*. The reason is mainly that the probability of the binary photon number detection reduces for large *N* as the state spreads in the Fock space after a displacement operation. However, this hybrid scheme beats the SNL as well, as indicated by the green region in Fig. 4c, with a maximum precision enhancement of 0.7 dB at *N* = 12. The obtained scaling *N*^−0.62^ (*N*^−0.69^ for an ideal experiment) is lower than that for the optimal scheme because of the sub-optimal detection process, but can still beat the standard scaling *N*^−0.5^ due to the initial MVS. Actually, by using a photon number resolving detector, which is available in optical domain, a better precision could be achieved by the hybrid scheme in future optical sensing applications (as shown by purple circles in Fig. 4c).

## Discussion

Our single-mode quantum metrology architecture achieves a precision near the HL and holds the advantage of hardware efficiency, minimized sensing configuration, and compatibility with quantum error correction that can be employed for further enhancement of the precision^43^. Our scheme can also be directly applied to other physical systems such as trapped ions^44^ and nitrogen-vacancy centers^45^. As demonstrated in the hybrid scheme, the precision still beats the SNL with the restricted detecting scheme consisting of only displacement operation and photon counting, which are easy to implement in optics. Additionally if we use microwave-to-optical up-conversion and down-conversion twice, near-HL precisions with the optimal-detecting scheme can be achieved. Our scheme thus also adds a powerful new platform to optical quantum metrology, which is quantum resource saving and robust compared to the multiple-path optical interferometer.

*Note added*: When submitting this work, we became aware of a related work which demonstrates the single phonon mode sensing using the superposition of Fock states in the trapped ion system^46^.

## Methods

### Device parameters

The superconducting system consists of two three-dimensional cavities and one ancillary transmon qubit, where the qubit couples with the two cavities simultaneously. The ancillary qubit has a frequency *ω*_q_/2*π* = 5.692 GHz, an energy relaxation time *T*_1_ = 30 μs, and a pure dephasing time *T*_*ϕ*_ = 120 μs. The short-lived cavity is at a frequency of *ω*_r_/2*π* = 8.610 GHz, has a lifetime of 44 ns due to its strong coupling to the external microwave drive line, and assists the fast high-fidelity readout of the ancillary qubit. The long-lived cavity serves as the sensing mode and has a frequency *ω*_s_/2*π* = 7.634 GHz, a single-photon lifetime $T_1^{\mathrm{s}} = 143\,\mu {\mathrm{s}}$, and a coherence time $T_2^{\mathrm{s}} = 250\,\mu {\mathrm{s}}$.

### Analysis of experimental imperfections

In practical quantum systems, there are inevitable imperfections, such as the decoherence of the ancillary qubit and the sensing mode, and the finite control precision. By including those imperfections in numerical simulations, we can estimate the phase estimation precision for practical experiments. It is worth noting that only the calibrated parameters of the system and the numerically optimized control pulses are used in the simulation, and neither further assumptions nor fitting parameters are introduced. The excellent agreement between the experimental and numerical results indicates that all essential experimental imperfections are captured in our numerical model and all experimental parameters are well calibrated. The numerical simulations further provide valuable information on the contribution of each imperfection to the loss of measurement precision. The detailed analysis of the errors in the three stages of the quantum metrology experiment: initial state preparation, evolution of the system during the sensing process, and final detection are described in Supplementary Note 3. Note that all the calculations and experiments are performed unconditionally with no post-selection of the experimental and numerical outcomes.

## Supplementary information

Supplementary Information

## Data Availability

The data that support the findings of this study are available from the corresponding authors upon reasonable request.

## Code availability

The code used for simulations is available from the corresponding authors upon reasonable request.
