# Supplementary material for: Heisenberg-limited single-mode quantum metrology in a superconducting circuit
Source: Nat Commun. 2019 Sep 26;10:4382. doi: 10.1038/s41467-019-12290-7 (PMC6763470; doi:10.1038/s41467-019-12290-7)
Supplement: Supplementary file 1 — Supplementary Information [file 41467_2019_12290_MOESM1_ESM.pdf]

Supplementary Information for: Heisenberg-limited single-mode quantum metrology in a superconducting circuit

W. Wang et al.

SUPPLEMENTARY NOTE 1 - SYSTEM HAMILTONIAN MODEL

Our superconducting system consists of one ancillary transmon qubit and two three-dimensional cavities, a short-lived one for fast high-fidelity readout of the qubit and a long-lived one for the sensing mode. For simplicity, we will refer to the long-lived cavity as the "cavity" or the "sensing mode" henceforth. Since the transition frequency of the qubit is largely off-resonant with the two cavity modes, the qubit nonlinearity would induce the dispersive couplings between the three components. In such a strong dispersive regime [1], the system can be described by the Hamiltonian

$$\begin{aligned} H_{\mathrm{qsr}}/\hbar = {} & \omega_{\mathrm{q}}|e\rangle\langle e| + \omega_{\mathrm{r}} a_{\mathrm{r}}{}^{\dagger} a_{\mathrm{r}} + \omega_{\mathrm{s}} a_{\mathrm{s}}{}^{\dagger} a_{\mathrm{s}} \\ & - \chi_{\mathrm{qs}}|e\rangle\langle e| a_{\mathrm{s}}{}^{\dagger} a_{\mathrm{s}} - \chi_{\mathrm{qr}}|e\rangle\langle e| a_{\mathrm{r}}{}^{\dagger} a_{\mathrm{r}} \\ & - \chi_{\mathrm{sr}} a_{\mathrm{s}}{}^{\dagger} a_{\mathrm{s}} a_{\mathrm{r}}{}^{\dagger} a_{\mathrm{r}}, \end{aligned} \tag{1}$$

where $\omega_{\mathrm{q,s,r}}$ are frequencies of the ancillary qubit, the sensing-mode cavity and the readout cavity, respectively; $a_{\mathrm{s,r}}$ are the corresponding bosonic operators; χ_{qs}, χ_{qr}, χ_{sr} are the cross-Kerr coefficients.

Considering that the readout cavity only functions when measuring the state of the ancilla and χ_{sr} is two orders of magnitude smaller than χ_{qs} and χ_{qr}, the Hamiltonian can be simplified by neglecting the readout cavity as

$$H_{\mathrm{qs}}/\hbar = \omega_{\mathrm{q}}|e\rangle\langle e| + \omega_{\mathrm{s}} a_{\mathrm{s}}{}^{\dagger} a_{\mathrm{s}} - \chi_{\mathrm{qs}}|e\rangle\langle e| a_{\mathrm{s}}{}^{\dagger} a_{\mathrm{s}}. \tag{2}$$

In such a dispersively coupled qubit-cavity system, the photonic state stored in the cavity could be manipulated through the qubit. On one hand, as demonstrated in previous works, when χ_{qs} is much larger than the decoherence rates of the qubit and the cavity, the photon number state could be resolved by probing the qubit transitions, or by a selective photon-number-dependent driving on the qubit [2]. Such a quantum non-demolition detection process allows high-fidelity state initialization of the cavity, and also enables the Fock state detection. On the other hand, arbitrary unitary operations of the cavity can be implemented with selective number-dependent arbitrary phase gates [3, 4] or optimal quantum control by gradient ascent pulse engineering [5, 6].

SUPPLEMENTARY NOTE 2 - THEORY

Fisher Information

In this work, we use Fisher information to quantify the phase sensitivity of our phase estimation measurement schemes. The general Fisher information is defined as [7]

$$F^{(N)} = \sum_n \left[\left(\frac{\partial \ln p_n^{(N)}}{\partial \theta} \right)^2 p_n^{(N)} \right], \tag{3}$$

where the label N corresponds to the photon number of the maximum variance state (MVS) $|\Psi(N)\rangle = (|0\rangle + |N\rangle)/\sqrt{2}$ used in the sensing schemes, θ is the parameters to be estimated, and $p_n^{(N)}$ is the probability for detecting n photons. According to the theory of parameter estimation, the precision is ultimately limited by the Cramér-Rao bound (CRB), which implies that

$$\delta\tilde{\theta} \geq \frac{1}{\sqrt{F^{(N)}}}. \tag{4}$$

The optimal scheme

As described in the main text, the optimal scheme is a Ramsey-like scheme that prepares the cavity in the MVS and projects the evolved state to MVS after the phase accumulation. In this case, the outcome is binary, as the ancillary qubit is projected to $|g\rangle$ or $|e\rangle$. In the main text, the measurement probability $P_{\mathrm{opt}}^{(N)}$ has a sensitivity with respect to the phase being measured θ as $\partial P_{\mathrm{opt}}^{(N)}/\partial\theta = -(N \sin N\theta)/2$, which oscillates with θ. To achieve the best precision, we can introduce a phase operation $U(-\varphi)$ associated with the detection operator to work at the most sensitive working point, with φ being an adjustable parameter. In our experiment, for the proof-of-principle demonstration of quantum enhanced phase measurement, both θ and φ are predetermined and thus we combine $U(\theta)$ and $U(-\varphi)$ together in the main text for simplicity. Below we present the more complete derivations of the precision of phase measurement.

In ideal experiments, we have the probability of measuring $|g\rangle$

$$P_{\text{opt}}^{(N)} = |\langle\Psi(N)|\,U(\theta)\,U(-\varphi)\,|\Psi(N)\rangle|^2 = \frac{1+\cos N(\theta-\varphi)}{2}, \tag{5}$$

where $U(\theta) = e^{i\theta a^\dagger a}$, and $U(-\varphi)\,|\Psi(N)\rangle = (|0\rangle + e^{-iN\varphi}\,|N\rangle)/\sqrt{2}$ is the initial MVS with a proper phase for the maximum Fisher information. According to Supplementary Equation 3, the Fisher information of the optimal scheme is

$$\begin{aligned} F_{\text{opt}}^{(N)} &= \frac{1}{P_{\text{opt}}^{(N)}}\left(\frac{\partial P_{\text{opt}}^{(N)}}{\partial\theta}\right)^2 + \frac{1}{1-P_{\text{opt}}^{(N)}}\left(\frac{\partial\left(1-P_{\text{opt}}^{(N)}\right)}{\partial\theta}\right)^2 \\ &= \left(\frac{1}{P_{\text{opt}}^{(N)}} + \frac{1}{1-P_{\text{opt}}^{(N)}}\right)\left(\frac{\partial P_{\text{opt}}^{(N)}}{\partial\theta}\right)^2 \\ &= \frac{1}{\left(1-P_{\text{opt}}^{(N)}\right)P_{\text{opt}}^{(N)}}\left(\frac{\partial P_{\text{opt}}^{(N)}}{\partial\theta}\right)^2. \end{aligned} \tag{6}$$

When $\varphi = \theta - \frac{\pi}{2N} - \frac{m\pi}{N}$ with m being an integer, the maximum achievable Fisher information reads

$$F_{\text{opt}}^{(N)} = N^2, \tag{7}$$

and the precision of the phase estimation [Supplementary Equation 4] is

$$\delta\tilde{\theta} \geq \frac{1}{N}. \tag{8}$$

This is the Heisenberg limit (HL) for a given mean photon number $N/2$.

In practical experiments, there are errors and decoherences during the operations and evolutions, thus the contrast of the measured Ramsey fringes reduces. Therefore, the measured probability reads

$$P^{(N)} = A + B\cos N(\theta-\varphi), \tag{9}$$

with $A = B = \frac{1}{2}$ for ideal experiments [Supplementary Equation 5]. Then, the maximum achievable Fisher information is

$$F_{\text{opt}}^{(N)} = \frac{1}{\left(1-P^{(N)}\right)P^{(N)}}\left(\frac{\partial P^{(N)}}{\partial\theta}\right)^2 = \frac{N^2B^2}{A\,(1-A)}, \tag{10}$$

and

$$\delta\tilde{\theta} \geq \frac{\sqrt{A\,(1-A)}}{NB}. \tag{11}$$

The hybrid scheme

The hybrid scheme is the sensing procedure to measure the photon number distribution after applying a displacement operation $D(\alpha)$ on the state $|\psi_0(N)\rangle = (|0\rangle + e^{iN\theta}|N\rangle)/\sqrt{2}$ with the accumulated phase. The cavity state before the photon number detection reads $|\psi(N,\alpha)\rangle = \sum_n c_n^{(N)}(\alpha)|n\rangle$, where

$$\begin{aligned} c_n^{(N)}(\alpha) &= \langle n|D(\alpha)|\psi_0(N)\rangle \\ &= \frac{e^{-|\alpha|^2/2}}{\sqrt{2}\sqrt{n!}}\left[\alpha^n + (-\alpha^*)^{N-n}e^{iN\theta}\frac{\sqrt{N!}}{(N-n)!}\,{}_1F_1(-n;N-n+1;|\alpha|^2)\right] \end{aligned} \tag{12}$$

for $0 \leq n \leq N$, and

$$c_n^{(N)}(\alpha) = \frac{e^{-|\alpha|^2/2}}{\sqrt{2}\sqrt{n!}}\left[\alpha^n + \alpha^{n-N}e^{iN\theta}\frac{n!}{(n-N)!\sqrt{N!}}\,{}_1F_1(-N;n-N+1;|\alpha|^2)\right] \tag{13}$$

for $n \geq N$. Here ${}_1F_1$ is the confluent hypergeometric function. Given these amplitudes, we can further compute the probability of measuring n microwave photons:

$$P_n^{(N)}(\alpha) = \frac{e^{-|\alpha|^2}}{2 \cdot n!} \Big\{ |\alpha|^{2n} + |\alpha|^{2(N-n)} \frac{N!}{[(N-n)!]^2} \left[{}_1F_1(-n; N-n+1; |\alpha|^2) \right]^2 + (-1)^{N-n} \frac{\sqrt{N!}}{(N-n)!} \cdot 2|\alpha|^N \cos[N(\theta - \varphi)] {}_1F_1(-n; N-n+1; |\alpha|^2) \Big\} \quad (14)$$

for $0 \leq n \leq N$, and

$$P_n^{(N)}(\alpha) = \frac{e^{-|\alpha|^2}}{2 \cdot n!} \Big\{ |\alpha|^{2n} + |\alpha|^{2(n-N)} \frac{(n!)^2}{[(n-N)!]^2 N!} \left[{}_1F_1(-N; n-N+1; |\alpha|^2) \right]^2 + \frac{n!}{(n-N)!\sqrt{N!}} \cdot 2|\alpha|^{2n-N} \cos[N(\theta - \varphi)] {}_1F_1(-N; n-N+1; |\alpha|^2) \Big\} \quad (15)$$

for $n \geq N$, where φ is the phase of $\alpha = |\alpha| e^{i\varphi}$.

For each n, the probability can be written as

$$P_n^{(N)} = A_n^{(N)} + B_n^{(N)} \cos N(\theta - \varphi), \quad (16)$$

where coefficients $A_n^{(N)}$ and $B_n^{(N)}$ can be directly derived from Supplementary Equations 14 and 15.

N	n_{opt}	$\lvert\alpha\rvert_{\mathrm{opt}}$	$\delta\tilde{\theta}^{(N)}_{\mathrm{opt\_ideal}}$	$\delta\tilde{\theta}^{(N)}_{\mathrm{opt\_sim}}$	$\delta\tilde{\theta}^{(N)}_{\mathrm{opt\_exp}}$
1	0	0.7178	1.1608	1.2354	1.2449
2	1	0.7654	0.7188	0.7567	0.7685
3	1	1.0000	0.5117	0.5404	0.5558
4	1	1.2128	0.4307	0.4590	0.4680
5	2	1.1493	0.3905	0.4219	0.4302
6	2	1.3207	0.3273	0.3549	0.3633
7	2	1.4854	0.2927	0.3267	0.3375
8	2	1.6412	0.2731	0.3129	0.3235
9	3	1.5571	0.2535	0.2924	0.3019
10	3	1.6969	0.2312	0.2696	0.2796
11	3	1.8322	0.2172	0.2563	0.2690
12	3	1.9619	0.2085	0.2532	0.2661
13	4	1.8773	0.1958	0.2506	
14	4	1.9983	0.1844	0.2607	

Supplementary Table 1. Numerical results of the optimal displacement amplitude ($|\alpha|_{\mathrm{opt}}$) and Fock state projection ($|n_{\mathrm{opt}}\rangle \langle n_{\mathrm{opt}}|$) for various N in the hybrid sensing scheme with binary detection. The corresponding precisions of the phase estimation $\delta\tilde{\theta}^{(N)}_{\mathrm{opt\_ideal}}$ for an ideal experiment without system decoherence and operation imperfections, and $\delta\tilde{\theta}^{(N)}_{\mathrm{opt\_sim}}$ with system decoherence and operation imperfections are also shown. $\delta\tilde{\theta}^{(N)}_{\mathrm{opt\_exp}}$ are measured data and already plotted in Fig. 4**c** of the main text.

Binary detection

In our experiment, we simulate the photon counting by projecting the state of the cavity to Fock state $|n\rangle$, thus obtain binary detection results for projections $\Pi_n = |n\rangle \langle n|$ and $\mathbb{I} - \Pi_n$ with $\mathbb{I}$ being the identity operator. Therefore, the maximum achievable

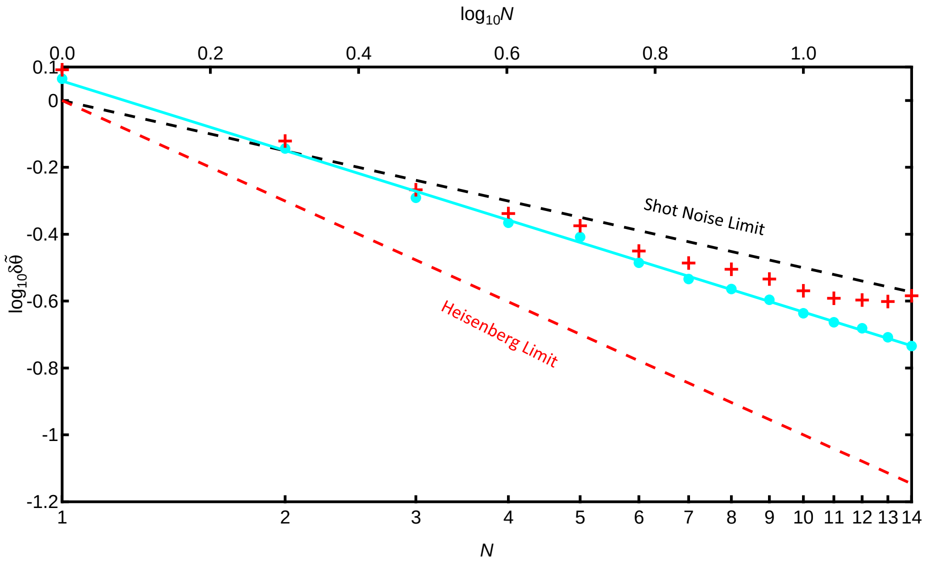

Supplementary Figure 1. Numerical results of the hybrid sensing scheme with binary detection. The red crosses are $\delta\tilde{\theta}^{(N)}_{\text{opt\_sim}}$ with system decoherence and operation imperfections in Supplementary Table 1, saturating at large N (≥ 12). Cyan dots are $\delta\tilde{\theta}^{(N)}_{\text{opt\_ideal}}$ in Supplementary Table 1 with no sign of saturation. The cyan solid line is a linear fit and gives $\delta\tilde{\theta}^{(N)}_{\text{opt\_ideal}} \sim N^{-0.69}$. The small fluctuation around the fit is due to the discreteness of n. The error bars, obtained through the standard deviations of $A_n^{(N)}$ and $B_n^{(N)}$ in fitting the simulated data as for Fig. 4**b** in the main text, are smaller than marker sizes.

Fisher information is

$$\begin{aligned} F_{\text{hyb}}^{(N)} &= \max_{n,\alpha}\left\{\frac{1}{P_n^{(N)}}\left(\frac{\partial P_n^{(N)}}{\partial\theta}\right)^2 + \frac{1}{1-P_n^{(N)}}\left(\frac{\partial\left(1-P_n^{(N)}\right)}{\partial\theta}\right)^2\right\} \\ &= \max_{n,\alpha}\left\{\frac{1}{\left(1-P_n^{(N)}\right)P_n^{(N)}}\left(\frac{\partial P_n^{(N)}}{\partial\theta}\right)^2\right\} \\ &= \frac{\left[NB_n^{(N)}\right]^2}{A_n^{(N)}\left(1-A_n^{(N)}\right)}, \end{aligned} \tag{17}$$

when $\varphi = \theta - \frac{\pi}{2N} - \frac{m\pi}{N}$ with m being an integer for the optimal n_{opt} and α_{opt}.

For our hybrid scheme, we numerically optimize n and $|\alpha|$ to calculate the maximum achievable Fisher information $F_{\text{hyb}}^{(N)}$. The numerical results with and without system decoherence and operation imperfections are summarized in Supplementary Table 1 and Supplementary Figure 1. The numerical results indicate that the uncertainty in the phase estimation measurement without system decoherence and operation imperfections by the hybrid scheme scales as $N^{-0.69}$, which beats the shot noise limit (SNL). The small fluctuation around the fit is due to the discreteness of n.

With current experimental conditions, the sensitivity of the hybrid sensing scheme with binary detection saturates at large N (≥ 12). The reasons are twofold. First, for large N, the deleterious effect from the system decoherence also increases. Second, when N gets bigger, a larger Hilbert space with more photon numbers are needed for the numerical optimization, whose fidelity will also decrease because the convergence of the optimization gets worse.

Photon number resolving detection

For future experiments in optical domain, it is possible to measure the photon number distribution of the output state $D(\alpha)U(\theta)|\Psi(N)\rangle$ by optical single-photon detectors. Therefore, the hybrid scheme is not restricted to the binary detection,

and better precision can be obtained by detecting the photon numbers Π_n for $n = 0, ..., n_{\text{trunc}}$, where n_{trunc} is the truncation of the photon counter. In this case, the maximum achievable Fisher information is

$$F_{\text{hyb}}^{(N)} = \max_{\alpha} \left\{ \sum_{n=0}^{n_{\text{trunc}}} \frac{1}{P_n^{(N)}} \left(\frac{\partial P_n^{(N)}}{\partial \theta} \right)^2 \right\}. \tag{18}$$

when $\varphi = \theta - \frac{\pi}{2N} - \frac{m\pi}{N}$ with m being an integer for the optimal α_{opt}.

In order to reveal the potential of the hybrid scheme, we numerically solve the maximum achievable Fisher information with $n_{\text{trunc}} = 90$. The numerical results without system decoherence and operation imperfections are summarized in Supplementary Table 2 and shown in Fig. 4**c** of the main text. The numerical results indicate that the uncertainty in the phase measurement by the hybrid scheme can be further improved by the photon-number-resolving detections, with an achievable precision scaling as $N^{-0.91}$ and approaching the HL.

N	$\lvert\alpha\rvert_{\text{opt}}$	$F_{\text{hyb}}^{(N)}$	$\delta\tilde{\theta}_{\text{opt\_ideal}}^{(N)}$
1	0.6970	0.7576	1.1489
2	0.9899	2.7602	0.6019
3	1.3248	5.7726	0.4162
4	1.1907	9.3633	0.3268
5	1.3966	14.5246	0.2624
6	1.6194	20.4490	0.2211
7	1.7879	26.2011	0.1954
8	1.7004	33.8945	0.1718
9	1.8777	42.4339	0.1535
10	2.0450	50.3537	0.1409
11	1.9634	59.9470	0.1292
12	2.1096	70.8052	0.1188

Supplementary Table 2. Numerical results of the optimal displacement amplitude ($\lvert\alpha\rvert_{\text{opt}}$) for photon-number-resolving detections in the hybrid sensing scheme with $n_{\text{trunc}} = 90$. The corresponding optimal precision of the phase estimation without system decoherence and operation imperfections $\delta\tilde{\theta}_{\text{opt\_ideal}}^{(N)} \sim N^{-0.91}$. These results have been plotted in Fig. 4**c** of the main text.

SUPPLEMENTARY NOTE 3 - ANALYSIS OF ERRORS

For an ideal system described by Supplementary Equation 2, the state preparation and detection could be implemented nearly perfectly by the optimal quantum control pulses. However, in practical experiments, there are imperfections that are not included in the ideal Hamiltonian, such as the decoherence of the qubit and the sensing mode, and the self-Kerr effect of the sensing mode. By including those imperfections in the numerical simulations, we can obtain the real phase estimation precision, and the results are provided in Figs. 3**c** and 4**c** in the main text. It is worth noting that only the calibrated parameters of the system and the control pulse envelope from our optimized control algorithm are used in the simulation, and no further assumptions are made. The excellent agreement between the experimental and numerical results indicates that our numerical model has included all essential imperfections in the experiments and all experimental parameters are well calibrated.

With the assistance of such an accurate numerical model, we analyze the origin of the errors in our quantum sensing schemes in this section. The errors induced by the experimental imperfections can be estimated by calculating the Fisher information loss (FIL), which quantifies the loss of precision due to these imperfections. The FIL is defined as

$$\text{FIL} = \frac{F_{\text{ideal}} - F_{\text{num}}}{F_{\text{ideal}}}, \tag{19}$$

where F_{ideal} is the maximum Fisher information expected from the ideal theory, and F_{num} is the maximum Fisher information from the results of numerical simulations including experimental imperfections.

The FIL for both optimal and hybrid schemes are summarized in Supplementary Table 3 and Supplementary Table 4, respectively. Here, the error sources are mainly divided into three parts: imperfect state preparation, imperfect evolution of the system during the sensing process, and imperfect detection. From the numerical results, we find that the imperfect initial state

Supplementary Table 3. Error budget for the optimal scheme.

Error sources	Assessment	Duration	FIL ($N = 1, 2, ..., 12$)
state initialization error	imperfect state preparation	700 ns	5.8%∼24.5%
infidelity in the evolution	qubit and cavity decoherence	528 ns	2%∼3%
gate error in U_H gate	parameter uncertainty and imperfect optimization	700 ns	∼1%
decoherence during U_H gate	qubit and cavity decoherence	700 ns	6.1%∼9.9%
qubit readout	qubit decoherence	320 ns	∼1%
total in experiment			11.0%∼32.2%

Supplementary Table 4. Error budget for the hybrid scheme.

Error sources	Assessment	Duration	FIL ($N = 1, 2, ..., 12$)
state initialization error	imperfect state preparation	700 ns	6.1%∼25.4%
pulse error in selective rotation	imperfect n selectivity	1.44 μs	<1%
decoherence during selective rotation	qubit and cavity decoherence	1.44 μs	4.2%∼9.5%
qubit readout	qubit decoherence	320 ns	∼1%
total in experiment			13.1%∼38.6%

preparation and errors in the detection process contribute most to the FIL. The state preparation error causes similar FIL for both schemes and increases with N. Although the detections are different in the two schemes, the resulted FILs are similar in the detection process with the dominant contributions from the qubit and cavity decoherence.

In the following, we provide detailed analysis of the errors in the three stages of the quantum metrology experiments: state preparation, evolution and detection. Note that all the calculations and experiments are performed unconditionally, i.e. there is no post-selection of the experimental and numerical outcomes.

Imperfections in state preparation and tomography

In the optimal scheme, the input initial state $|e\rangle(|0\rangle + |N\rangle)/\sqrt{2}$ is prepared with optimal control pulses with a duration of 700 ns. During this period, there is decoherence of the qubit and the sensing mode, causing loss of fidelity of the initial states. The decay rate of the photons in the sensing mode is proportional to photon number. Preparation of states $(|0\rangle + |N\rangle)/\sqrt{2}$ with a large photon number N entails high probability of populating the cavity in the high energy levels (could be much higher than N) during the optimal control pulses, leading to larger loss of the initial state fidelity for a larger N.

To infer the imperfection during the state preparation, we measure the probability of the qubit state being in $|e\rangle$ after the state preparation process. The results are summarized in Supplementary Table 5, showing the probability of ending up with the state $|g\rangle(|0\rangle + |N\rangle)/\sqrt{2}$ is less than 0.03.

In the experiment, we characterize the generated initial states through the Wigner functions. The state fidelity is calculated as $F = \langle\Psi(N)|\,\rho_m\,|\Psi(N)\rangle$, where ρ_m is the reconstructed density matrix of the measured state in the experiment. The experimental results are provided in Supplementary Table 6. Due to a displacement pulse with a length of 20 ns before the Wigner function measurement, the cavity states obtain extra phases proportional to the photon number N. Compensating the relative phase between $|0\rangle$ and $|N\rangle$, the fidelities of reconstructed initial states are 0.92, 0.92, 0.90, 0.91, 0.90, 0.88, 0.85, 0.83, 0.81, 0.80, 0.72, 0.70 for $N = 1$ to 12, respectively.

Through numerical simulations, we firstly calculate the fidelities of the generated state based on the optimal control pulses directly. The results are calculated also with proper phase compensations. However, the fidelities are much higher than the measured ones. We attribute the discrepancy to the imperfect tomography and reconstruction process. We next perform a full numerical simulation including system decoherence and the Wigner tomography process (identical to the experimental sequence), and the obtained fidelities then agree well with the experiment. All results are shown in Supplementary Table 6. A detailed discussion on the errors in the tomography process can be found in Ref. [2]. Finally, we estimate the FIL due to the state initialization to be 5.8% $\sim$ 24.5% for the optimal scheme. The numbers for the hybrid scheme are about the same.

N	Experiment	Simulation
1	0.9805	0.9876
2	0.9778	0.9879
3	0.9789	0.9872
4	0.9784	0.9882
5	0.9778	0.9906
6	0.9752	0.9883
7	0.9743	0.9891
8	0.9773	0.9884
9	0.9758	0.9874
10	0.9748	0.9850
11	0.9751	0.9859
12	0.9722	0.9844

Supplementary Table 5. Excited state population of the qubit in the experiment and in the simulation in the optimal scheme for different initial target states $|e\rangle\,(|0\rangle+|N\rangle)/\sqrt{2}$.

N	Experiment reconstructed	Simulation angle adjusted	simulation whole process
1	0.92	0.99	0.96
2	0.92	0.98	0.96
3	0.90	0.98	0.96
4	0.91	0.98	0.95
5	0.90	0.98	0.95
6	0.88	0.98	0.95
7	0.85	0.97	0.89
8	0.83	0.97	0.88
9	0.81	0.95	0.86
10	0.80	0.92	0.84
11	0.72	0.91	0.75
12	0.70	0.88	0.76

Supplementary Table 6. Measured and simulated fidelities of the sensing mode initial state $(|0\rangle+|N\rangle)/\sqrt{2}$ in the optimal scheme.

Imperfections during the evolution

In real quantum metrology applications, there is a time interval for the sensing mode to interact with the entity under detection to acquire the necessary information. In our experiment, we just wait for certain time to accumulate a phase in the optimal scheme, or directly combine the phase into the following displacement operations in the hybrid scheme. For the optimal scheme, the waiting time of 528 ns is not negligible compared to the decoherence time of the qubit, thus contributes $2\% \sim 3\%$ to FIL. While for the hybrid scheme, the displacement operation is short enough that we can ignore its contribution to FIL.

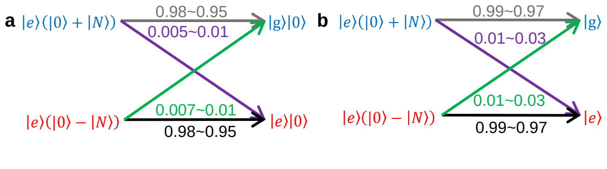

Supplementary Figure 2. The errors of the quasi-Hadamard gate U_H due to the system decoherence (**a**) and the errors by further considering the cavity photon-number-independent readout (**b**). Note that the decoherence during the gate U_H leads to information leakage out to the external space, i.e. the states $|g\rangle|n\rangle$ with $n \neq 0$.

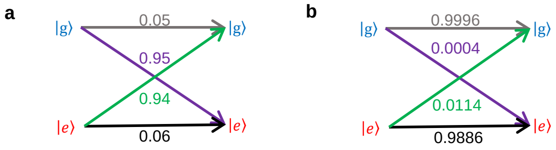

Supplementary Figure 3. The performance of the selective π rotation pulse R_π^n (**a**) and the qubit readout (**b**) in the experiment.

Imperfections in the detection

Optimal scheme

In the optimal scheme, the detection comprises two processes: (i) the quasi-Hadamard gate U_H for the Ramsey-like interference, and (ii) the readout of the qubit state. In the process (i), an optimal control pulse of 700 ns is implemented, which converts $|e\rangle(|0\rangle+|N\rangle)/\sqrt{2}$ to $|g\rangle|0\rangle$ and $|e\rangle(|0\rangle-|N\rangle)/\sqrt{2}$ to $|e\rangle|0\rangle$, i.e. decodes the two MVSs onto the state of the qubit. In the process (ii), the qubit state is readout with the assistance of the readout cavity within a duration of about 320 ns.

The imperfections mainly contribute in three aspects:

- The optimized control pulse may not be perfectly implemented in the experiments due to the uncertainty of the system parameters and the control circuit. Besides, the control pulse is not fully optimized numerically for obtaining a perfect U_H. We expect this gate-error-induced FIL $\sim 1\%$.

- The decoherence of the qubit and the cavity. During the process (i), the cavity might be pumped to Fock states with large photon numbers, and the qubit to the excited state, thus the decay and dephasing effect would reduce the fidelity of the operation. The numerical analysis with decoherence for gate fidelities of U_H is shown in Supplementary Figure 2**a**. In process (ii), only the qubit state is projectively measured, while the cavity state is not. However, the decoherence during the gate U_H leads to information leakage out to the external space, i.e. the states $|g\rangle|n\rangle$ with $n\neq 0$ are also included in the outcome of $|g\rangle$. As shown in Supplementary Figure 2**b**, such processes increase the errors. From numerical simulations, the FIL due to the qubit and cavity decoherence during U_H gate is found to be 6.1%∼9.9% for $N=1,2,...12$.

- Wrong readout. For the process (ii), the qubit in the excited state $|e\rangle$ might decay and leads to wrong detection outcomes. The readout fidelity is 0.9996 for$|g\rangle$ and 0.9886 for $|e\rangle$, as shown in Supplementary Figure 3**b**. This readout infidelity contributes about 1% FIL.

Hybrid scheme

In the hybrid scheme, the detection comprises three processes: (i) the displacement operation on the cavity state, (ii) the photon number selective rotation pulse on the qubit, (iii) the readout of the qubit state. In the process (i), the duration is so short that the imperfection can be neglected. For the process (iii), the error is the same as that in the optimal scheme. For the process (ii), there are two main contributions:

- The imperfect photon number selective pulse. The qubit selective π operation R_π^n in this scheme (Fig. 4 in the main text) requires narrow enough bandwidth in order to distinguish the target photon number state $|n\rangle$ of the cavity from other photon numbers. The frequency difference for R_π^n and $R_\pi^{n\pm1}$ corresponds to the dispersive coupling strength $\chi_{\mathrm{qs}}/2\pi = 1.9$ MHz. We have used Gaussian pulses with a duration of 1.44 μs for the qubit selective rotations, which gives a spectral bandwidth of $\sigma_\omega/2\pi = 440$ kHz, not significantly smaller than χ_{qs}. Therefore, the photon number selective pulse would lead to wrong outcome by exciting the qubit when the cavity state is not $|n\rangle$. The performance of R_π^n is summarized in Supplementary Figure 3**a**. From our numerical simulation, the imperfect spectral selectivity would contribute $<1\%$ FIL.

- The system decoherence. During the 1.44 μs photon number selective excitation, the decoherence of the qubit and cavity would also degrade the fidelity of the operation. According to the measured decoherence, the conversion inefficiency of the π pulse is 4.8%, which causes 4.2%∼ 9.5% of FIL for $N=1,2...12$.

SUPPLEMENTARY REFERENCES

[1] Devoret, M. H. & Schoelkopf, R. J. Superconducting circuits for quantum information: An outlook. *Science* **339**, 1169–1174 (2013).
[2] Wang, W. *et al.* Converting quasiclassical states into arbitrary fock state superpositions in a superconducting circuit. *Phys. Rev. Lett.* **118**, 223604 (2017).
[3] Krastanov, S. *et al.* Universal control of an oscillator with dispersive coupling to a qubit. *Phys. Rev. A* **92**, 040303 (2015).
[4] Heeres, R. W. *et al.* Cavity state manipulation using photon-number selective phase gates. *Phys. Rev. Lett.* **115**, 137002 (2015).
[5] Khaneja, N., Reiss, T., Kehlet, C., Schulte-Herbrüggen, T. & Glaser, S. J. Optimal control of coupled spin dynamics: design of NMR pulse sequences by gradient ascent algorithms. *J. Magn. Reson.* **172**, 296 – 305 (2005).
[6] de Fouquieres, P., Schirmer, S., Glaser, S. & Kuprov, I. Second order gradient ascent pulse engineering. *J. of Magn. Reson.* **212**, 412 – 417 (2011).
[7] Paris, M. G. Quantum estimation for quantum technology. *Int. J. Quantum Inf.* **7**, 125–137 (2009).
